# Synthesis and Biological Evaluation of Novel Imidazole Derivatives as Antimicrobial Agents

**DOI:** 10.3390/biom14091198

**Published:** 2024-09-23

**Authors:** Huda A. Al-Ghamdi, Fahad A. Almughem, Manal A. Alshabibi, Abrar A. Bakr, Abdullah A. Alshehri, Alhassan H. Aodah, Nourah A. Al Zahrani, Essam A. Tawfik, Laila A. Damiati

**Affiliations:** 1 Department of Chemistry, College of Science, University of Jeddah, Jeddah 23218, Saudi Arabia; halgamdi4@uj.edu.sa (H.A.A.-G.); nalzahrani2@uj.edu.sa (N.A.A.Z.); 2 Advanced Diagnostics and Therapeutics Institute, Health Sector, King Abdulaziz City for Science and Technology (KACST), Riyadh 11451, Saudi Arabia; falmughem@kacst.gov.sa (F.A.A.); malshabibi@kacst.gov.sa (M.A.A.); aabakr@kacst.gov.sa (A.A.B.); abdualshehri@kacst.gov.sa (A.A.A.); aaodah@kacst.gov.sa (A.H.A.); 3 Department of Biological Science, College of Science, University of Jeddah, Jeddah 23218, Saudi Arabia

**Keywords:** Imidazole derivative, biological activity, antimicrobial activity, cell viability, chemical synthesis

## Abstract

Imidazole derivatives are considered potential chemical compounds that could be therapeutically effective against several harmful pathogenic microbes. The chemical structure of imidazole, with a five-membered heterocycle, three carbon atoms, and two double bonds, tends to show antibacterial activities. In the present study, novel imidazole derivatives were designed and synthesized to be evaluated as antimicrobial agents owing to the low number of attempts to discover new antimicrobial agents and the emerging cases of antimicrobial resistance. Two imidazole compounds were prepared and evaluated as promising candidates regarding in vitro cytotoxicity against human skin fibroblast cells and antimicrobial activity against several bacterial strains. The synthesized imidazole derivatives were chemically identified using nuclear magnetic resonance (NMR) and Fourier-transform infrared spectroscopy (FTIR). The results demonstrated a relatively high cell viability of one of the imidazole derivatives, i.e., HL2, upon 24 and 48 h cell exposure. Both derivatives were able to inhibit the growth of the tested bacterial strains. This study provides valuable insight into the potential application of imidazole derivatives for treating microbial infections; however, further in vitro and in vivo studies are required to confirm their safety and effectiveness.

## 1. Introduction

In the last century, nitrogen-based heterocycles attracted huge attention owing to their core scaffold that demonstrates promising pharmacological properties and therapeutic applications across various disease areas. The versatility of nitrogen heterocycles in medicinal chemistry led to the development of numerous drugs, including antivirals, and their ability to form hydrogen bonds with biological targets has made them valuable building blocks for the design of new drug candidates [1,2,3,4]. Imidazole’s derivatives are compounds that contain a five-membered ring with two nitrogen atoms and are widely used in different fields, including medicine. Among the various chemical compounds explored in medicinal chemistry, imidazole derivatives have attracted significant attention due to their versatile pharmacological properties [5]. Furthermore, the electron-rich nitrogen heterocycle is capable of readily accepting or donating a proton and can easily establish a variety of weak interactions [6]. These intermolecular forces, such as hydrogen bonding, dipole–dipole interactions, hydrophobic effects, van der Waals forces, and π-stacking interactions, have increased the significance of nitrogen compounds in medicinal chemistry. These interactions allow nitrogen compounds to bind with a wide range of enzymes and receptors in biological targets with high affinity owing to their enhanced solubility. The structural features of their derivatives are particularly advantageous, as they exhibit a broad spectrum of bioactivities [4,6,7]. 

Imidazole derivatives, such as 1H-imidazole, are widely used in the treatment of various diseases, showcasing their versatility and importance in medicinal chemistry. They are involved in combating conditions like cancer, bacterial infections, fungal infections, viral diseases, inflammation, diabetes, and Alzheimer’s disease. The broad range of biological activities exhibited by imidazole compounds underscores their significance in drug development and therapeutic interventions across multiple disease areas [8,9,10,11,12]. For instance, the applications of imidazole ring core scaffolds have exhibited potential anti-cancer, anti-bacterial, and anti-inflammatory effects [13]. 

The structural characteristics of imidazole derivatives enhance their ability to establish multiple drug–ligand interactions. The anticancer potential of imidazole derivatives can be attributed to their ability to inhibit cancer cell proliferation by interfering with key cellular processes [14]. These chemical compounds could target the enzymes involved in the DNA replications and repair that are leading to DNA damage and cell death by inducing apoptosis (programmed cell death) in cancer cells [15]. Also, imidazole derivatives have shown promising results in preclinical studies as potential anticancer agents. For example, 2-phenylimidazo [1,2-a]pyridine (PIP) has been shown to inhibit the growth of various cancer cell lines by inducing apoptosis [16]. Similarly, 4-(4-methylpiperazin-1-yl)-N-[3-(trifluoromethyl)phenyl]benzamide (MI-63) has demonstrated potent antitumor activity in animal models [17]. In addition to their therapeutic potential, imidazole derivatives have also been used as diagnostic tools in medical imaging. Radioactive isotopes can be attached to imidazole molecules and used to visualize tumors or other abnormal tissues in the body [18].

In terms of antibacterial and antifungal activities, imidazole derivatives demonstrated a potential effect to inhibit the growth of several bacterial strains, including Gram-positive and Gram-negative bacteria as well as *Candida albicans* [5]. Their mechanism of action involves interfering with bacterial DNA replication, cell wall synthesis, and cell membrane disruption [19,20,21]. Therefore, in the current study, we aim to synthesize new imidazole derivatives and investigate their effects on the growth of human skin fibroblasts and their antimicrobial activity against *Staphylococcus aureus* and *Escherichia coli*, *Pseudomonas aeruginosa*, and *Acinetobacter baumannii*. This work seeks to provide further insights into the potential of these new compounds in both therapeutic and antimicrobial contexts.

## 2. Materials and Methods

### 2.1. Materials

The analytical solvents used in the synthesis were provided by both Sigma Aldrich and Fisher. The prepared imidazole compounds were separated using column chromatography on silica gel (0.063–0.2 mm), and the products’ purity was verified by thin-layer chromatography (TLC) using an aluminum silica gel F254, with the spot being identified using iodine. Vancomycin HCl was provided by Tabuk Pharmaceuticals (Lot number 76491; Riyadh, Saudi Arabia) and ciprofloxacin was purchased from Sigma Aldrich (Saint Louis, MO, USA). All bacterial strains were purchased from the American Type Culture Collection (ATCC) (Manassas, VA, USA) as reference bacteria. Microbial strains of *Staphylococcus aureus* (ATCC 29213), methicillin-resistant *Staphylococcus aureus* (MRSA; ATCC 43300), *Escherichia coli* (ATCC 25922)*, Pseudomonas aeruginosa* (ATCC 1744), and *Acinetobacter baumannii* (ATCC 747) were used. Mueller–Hinton agar (MHA) and Mueller–Hinton broth (MHB) were obtained from Sigma-Aldrich (Taufkirchen, Germany) and prepared according to the manufacturer’s instructions.

### 2.2. Synthetic Procedures of Imidazole Derivatives (HL1 and HL2)

**Scheme 1:**
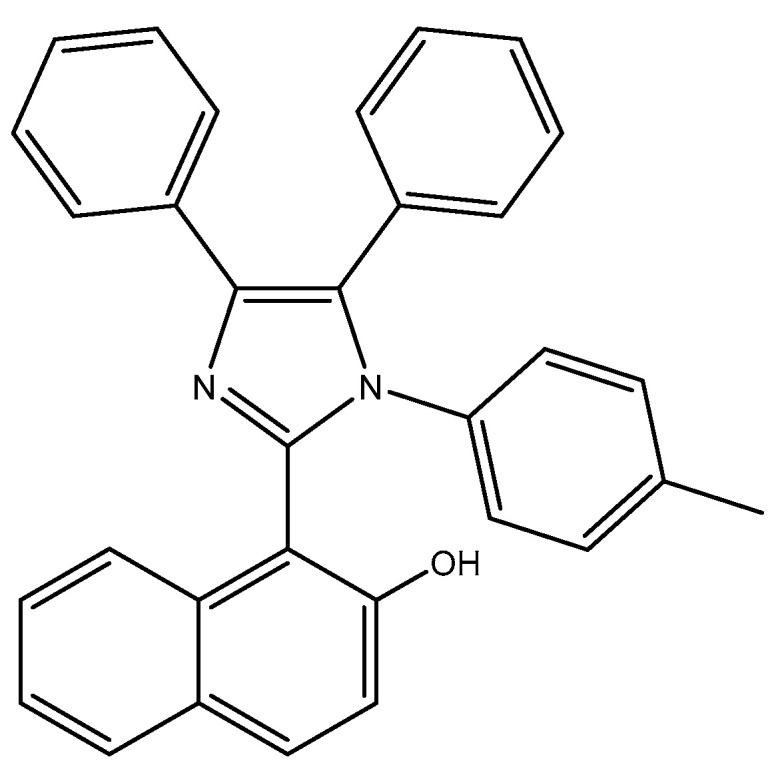
1-(4,5-diphenyl-1-(p-tolyl)-1H-imidazol-2-yl) naphthalen-2-ol, (HL1).

**Scheme 2:**
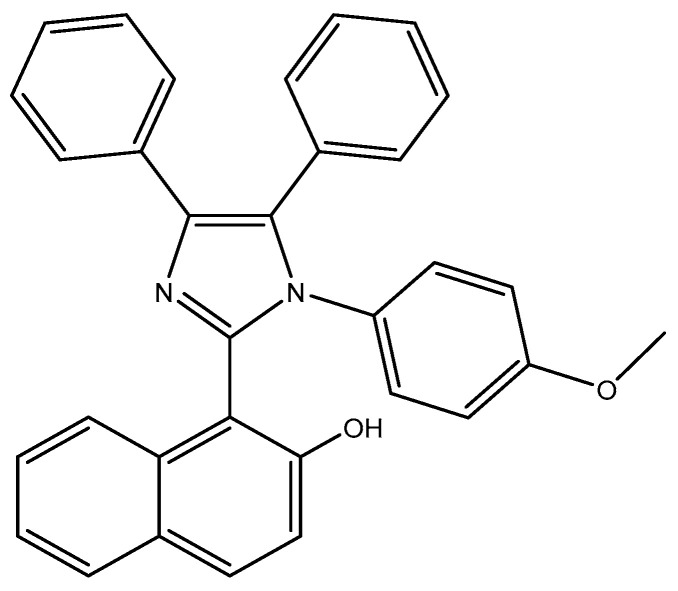
1-(1-(4-methoxyphenyl)-4,5-diphenyl-1H-imidazol-2-yl) naphthalen-2-ol, (HL2).

A solution of 2-Hydroxy-1-naphthaldehyde (0.122 g, 1 mmol, benzil (0.231 g, 1.1 mmol), 4-methylaniline (0.492 g, 4 mmol) or 4-methoxyaniline (0.428 g, 4 mmol), and ammonium acetate (0.77 g, 10 mmol) in glacial acetic acid (10 mL) was refluxed under argon for 12 h. After this exposition time, the reaction mixture was cooled and diluted with water (20 mL), and the solid formed was filtered off. The collected precipitate was washed with 10% acetic acid (4 × 5 mL) and water and dried to obtain the pure product of HL1 and HL2 (Scheme 1 and Scheme 2).

### 2.3. Nuclear Magnetic Resonance (NMR) Analysis

The NMR spectra were recorded in DMSO–d6 on Bruker Avance (Bruker Biospin, Rheinstetten, Germany) 600 MHz spectrometers for 1HNMR 32 scans and 500 MHz 2K scans for ^13^CNMR. The chemical shifts (δ) were reported in ppm and coupling constants in Hz.

### 2.4. Fourier-Transform Infrared Spectroscopy (FTIR) Analysis

FTIR spectra were recorded using a ThermoScientific Nicolet iS10 FTIR spectrometer (Thermo Fisher Scientific, Waltham, MA, USA). The synthesized imidazole derivatives were placed as KBr discs and were conducted at a wavenumber range of 4000 to 500 cm^−1^.

### 2.5. X-ray Diffraction (XRD) Analysis

The solid-state of imidazole derivatives was characterized using a Rigaku Miniflex 300/600 (Tokyo, Japan) equipped with Cu Kα radiation (λ = 1.5148227 Å). The specimen was mounted directly on a glass slide and examined at 40 kV and 15 mA, with a scan speed of 5°/minute over a range of 2θ 2° to 60°. The results were plotted by OriginPro 2024 (Origin Lab Corporation, Northampton, MA, USA).

### 2.6. Cell Viability Study

Human skin fibroblasts (HFF-1) were purchased from the American Type Culture Collection (ATCC) under catalogue number SCRC-1041 (Manassas, VA, USA). The cell line was routinely cultured and maintained in high glucose Dulbecco’s Modified Eagle Medium (DMEM) for HFF-1 cell line, fetal bovine serum (FBS), and antibiotic solution, which were all bought from Sigma-Aldrich (St. Louis, MO, USA). The antiproliferative activity assessment of tested compounds was conducted using the MTS assay (CellTiter 96^®^ aqueous cell proliferation one solution), which was bought from Promega (Southampton, UK).

The imidazole derivatives (HL1 and HL2) were prepared by dissolving in 10% *w*/*v* DMSO and then completed with fresh cell culture media to prepare a stock solution of 10,000 µg/mL. Then, 8 serial dilutions were assessed, starting from 5000 µg/mL down to 39 µg/mL. The cell viability was evaluated by cell titer 96^®^ aqueous one solution cell proliferation assay (MTS kit; Promega, UK). Briefly, HFF-1 cells were seeded into 96-well plates with a density of 1.5 × 10^4^ cells per well. Cells were incubated in a cell culture incubator at 37 °C overnight. After cells adhered, the HL1 and HL2 were added with different concentrations, as explained before, for 24 and 48 h at 37 °C. Negative control (DMEM only) and positive control (0.2% triton x-100) were used as experimental controls. After 24 and 48 h, cells were washed with sterile phosphate-buffered saline (PBS, pH 7.4), and then, 100 µL of fresh complete DMEM medium was added with 20 µL of MTS reagent per well. Cells were then incubated for 3 h in a cell culture incubator. The absorbance at 492 nm was recorded using a Cytation 3 absorbance microplate reader (BIOTEK Instruments Inc., Winooski, VT, USA). The percentage of cellular viability was calculated using the following equation:

Cellular Viability (%) = (S − T)/(H − T) × 100

where S is the absorbance of the cells treated with the tested compounds, T is the absorbance of the positive control, and H is the absorbance of the negative control.

### 2.7. Antibacterial Activity Study

The broth microdilution method was used to determine the minimum inhibitory concentration (MIC) values of the imidazole derivatives (HL1 and HL2), and vancomycin and ciprofloxacin as experimental controls, following the modified CLSI reference method [22]. The imidazole derivatives and ciprofloxacin were prepared in 10% DMSO, while vancomycin was dissolved in distilled water. Both derivatives were diluted in MHB by twofold serial dilution starting from 5000 to 2.44 μg/mL. However, the antibiotics were serially diluted at a concentration range of 40–0.02 μg/mL and added to the 96-well plates. Then, single pure colonies of bacteria were used to prepare the inoculums and adjusted to 0.5 McFarland standard giving a cell density of 1.5 × 10^8^ CFU/mL. A 100 μL solution was then added to each well, giving a final inoculum of 1.5 × 10^5^ CFU/mL cell density. The inoculated wells contained only bacteria used as growth control, and uninoculated wells contained only media (i.e., MHB) were used as a negative control. The 96-well plates were incubated overnight at 37 °C with a continuous shaking speed of 160 RPM. The lowest concentration with no visible growth and turbidity was considered as the MIC, and the bacterial growth inhibition was measured at a UV absorbance of 600 nm using a PowerWave XS2 plate reader (bioMérieux, Marcy L’Etoile, France). The background effect of the uninoculated wells was used to subtract the color of the derivatives in the treated wells.

### 2.8. Statistical Analysis

Experiments were performed as 3 independent replicates, and the results were expressed as the mean ± standard deviation (SD) and calculated using GraphPad Prism v.10.

## 3. Results and Discussion

### 3.1. Chemical Profile of Imidazole Derivatives

**Scheme 3:**
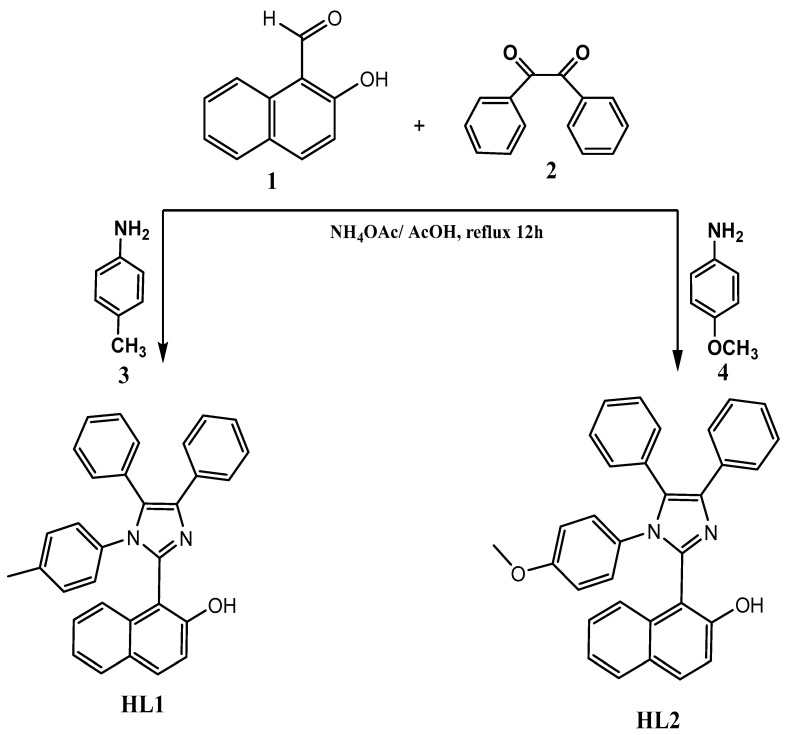
Synthesis of imidazole derivatives, HL1 and HL2.

The preparation of the new imidazole derivatives, HL1 and HL2, is shown in Scheme 3. The Debus–Radziszewski reaction was used to synthesized the derivatives. 2-Hydroxy-1-naphthaldehyde (1) reacted with benzil (2) and 4-methylaniline (3) or 4-methoxyaniline (4) in the presence of ammonium acetate in refluxing glacial acetic acid to afford imidazoles (HL1 and HL2). The chemical structure of two compounds was confirmed by IR, ^1^HNMR [23].

The infrared spectrum of HL1 (Supplementary Figure S1) and HL2 (Supplementary Figure S4) displayed certain distinctive stretching bands corresponding to the functional groups -C=C- and -C=N- that are located at 1513–1589 and 1602−1626 cm^−1^. The existence of aromatic C-H stretch is indicated by the stretching frequency at 3040–3058 cm^−1^. A further ^1^HNMR spectra reveal the presence of aromatic protons in the 6–8 ppm region, which is equivalent to 20 protons in the generated compounds (Supplementary Figure S2 for HL1 and Supplementary Figure S5 for HL2). The ^13^CNMR spectra represent aromatic C at 109.67–137.67 ppm, C=N at 143.42, 143.87 ppm, and C-OH at 154.94, 154.95 ppm. This validates the structure given to the target molecules (Supplementary Figure S3 for HL1 and Supplementary Figure S6 for HL2) [24].

The XRD results that are shown for HL1 and HL2 (Supplementary Figure S7) exhibited the crystalline nature of these derivatives. This observation is consistent with Volkov et al. (2022) [25] and Kowalkowska et al. (2017) [26], whose synthesized imidazole derivative products were crystalline. Volkov et al. (2022) synthesized their imidazole derivative by mixing thiourea with imidazole or 2-methylimidazole dissolved in methanol, whereas Kowalkowska et al. (2017) synthesized their imidazole derivative by mixing 1-(3-aminopropyl)-imidazole or 1,4-bis(imidazol-1-yl)-butane with either acetylacetonates or silanethiolates.

It is assumed that the addition of 2-Hydroxy-1-naphthaldehyde to the imidazole can enhance the antibacterial activity of the imidazole derivatives that were synthesized. It was reported that 2-hydroxy-1-naphthaldehyde was used to synthesize a Schiff base that consists of 2-hydroxy-1-naphthaldehyde and 2-amino-3-methylpyridine, which were complexed with different metal ions, and the results show that this product is effective against different bacterial strains such as *Staphylococcus aureus* and *Escherichia coli* [27]. Therefore, the cytotoxicity and antibacterial activities of HL1 and HL2 were evaluated.

### 3.2. Cell Viability Finding

To assess the two imidazole derivatives’ (HL1 and HL2) effect on the cellular metabolic activity of human dermal fibroblasts, i.e., HFF-1 cells, following their incubation for 24 and 48 h, the MTS assay was performed. Since the synthesized derivatives are new, a cell viability assay was performed on HFF-1 as a representative cell line to evaluate the safety of these novel derivatives. The HFF-1 cell line comprises susceptible cells that can be easily affected by any source of cytotoxicity that might be induced by the applied materials. Moreover, the HFF-1 cells are commonly used to assess the cytotoxicity of new drugs or novel compounds. In addition, two time points (24 and 48 h cell exposure) were used in this assessment, to evaluate the effect of exposure time on cell viability.

**Figure 1:**
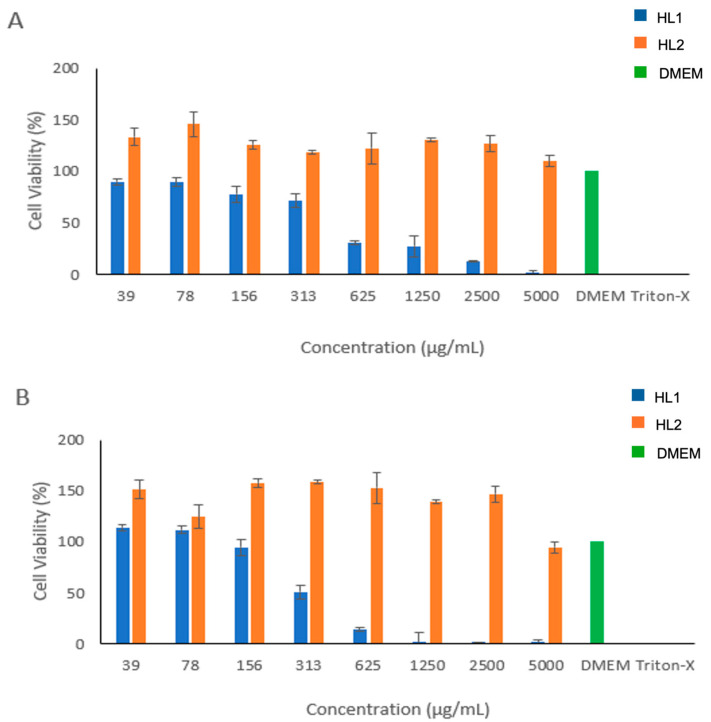
Cell viability of HFF-1 cells with different concentrations of imidazole derivatives (HL1 and HL2) at 24 h (**A**) and 48 h (**B**). These data are the MTS assay results, which are expressed as cellular viability (%) and presented as the mean ± SD (*n* = 3). DMEM was used as a negative control, while 0.2% triton x-100 as a positive control.

The result of the MTS assay following the incubation of HL1 and HL2 for 24 h is presented in Figure 1A. The application of HL1 for 24 h exhibited that the lowest used concentrations (39 and 78 µg/mL) demonstrated high cellular metabolic activity (≥80%) of HFF-1 cells, as shown in Figure 1A, while increasing the concentration to >313 µg/mL showed a significant reduction in the cellular viability to less than 50%. Conversely, the exposure of HL2 derivative to HFF-1 cells for 24 h exhibited high cell viability at all tested concentrations (5000 to 39 µg/mL), as shown in Figure 1A.

The effect of HL1 and HL2 compounds upon incubation with HFF-1 cells for 48 h was also evaluated and the result of the MTS assay was presented in Figure 1B. Applying concentrations of ≤156 µg/mL of HL1 derivative exhibited high cell viability (≥80%), whereas the doses of ≥313 µg/mL showed a significant reduction in the cell viability of HFF-1 cells. However, the application of the HL2 compound showed no reduction in the cell viability at all tested concentrations (5000 to 39 µg/mL), as demonstrated in Figure 1B.

The derivatives of 2-(4-aminophenyl)-1H-imidazo[4,5-f][1,10]phenanthroline with different unprotected monosaccharides have been studied by Gratal et al. and the result showed significant antiproliferative activity against tumor cell lines HeLa, PC3, and to a lesser extent, MCF7 and normal fibroblast cells (HFF1), with a better effect than the chemotherapy agent, cisplatin [28]. It has been reported that the application of benzene sulfonamide-bearing imidazole derivatives containing 4-chloro and 3,4-dichloro substituents in the benzene ring and 2-ethylthio and 3-ethyl groups in the imidazole ring on cancerous cells have antitumor activity against human triple-negative breast cancer MDA-MB-231 and human malignant melanoma IGR39 cell lines [29]. Furthermore, nine piperazine-tagged imidazole derivatives were evaluated on five different cell lines, MCF-7, PC3, Du145, HepG2, and HFF-1, and two of them were the most potent anticancer agents on HepG2 and MCF-7 cells with a minimal effect on HFF-1 cells [30]. Therefore, the synthesized HL2 imidazole derivative in this current study demonstrated a non-toxic effect on the conducted cells upon the applied concentrations (≤5000 µg/mL).

### 3.3. Antibacterial Activity Finding

The antibacterial activity of the synthesized imidazole derivatives was evaluated against the Gram-positive strains of *Staphylococcus aureus* (ATCC 29213) and MRSA (ATCC 43300) and Gram-negative strains of *Escherichia coli* (ATCC 25922), *Pseudomonas aeruginosa* (ATCC 1744), and *Acinetobacter baumannii* (ATCC 747). The strains were treated with different concentrations of imidazole derivatives ranging from 5000 to 2.44 μg/mL (Supplementary Figure S8). Vancomycin and ciprofloxacin were included in the study as reference antibiotics at a concentration range of 40–0.02 μg/mL (Supplementary Figure S9). Table 1 shows the MIC values of the imidazole derivatives and the reference antibiotics against all tested bacterial strains. For the Gram-positive strains, HL1 exhibited a MIC of 625 μg/mL and 1250 μg/mL against *Staphylococcus aureus* and MRSA, respectively, while HL2 showed inhibition at 625 μg/mL for both strains. In Gram-negative bacterial strains, the HL2 had a higher MIC than the Gram-positive strains, which valued 2500 μg/mL against *Escherichia coli, Pseudomonas aeruginosa*, and *Acinetobacter baumannii.* Moreover, HL1 displayed a higher MIC compared to HL2 against *Acinetobacter baumannii* (1250 μg/mL) and *Pseudomonas aeruginosa* (5000 μg/mL) but no inhibition was observed against *Escherichia coli* (i.e., >5000 μg/mL). The MICs of the standard antibiotics, i.e., vancomycin and ciprofloxacin, were 10–0.02 μg/mL against the tested bacterial strains. Nevertheless, vancomycin was not active at the tested concentration of 40 μg/mL against *Pseudomonas aeruginosa* or *Acinetobacter baumannii.*

Previous studies demonstrated that several imidazole derivatives might inhibit the growth of bacteria by disrupting the cell wall synthesis or inhibiting the protein synthesis. A study by Brahmbhat et al. evaluated the effect of 3-(2-4-diphenyl-1H-imidazole-z-y)-1H-pyrazole on *Staphylococcus aureus, Pseudomonas aeruginosa, Escherichia coli*, and *Bacillus subtilis*. The study concluded that the derivative showed a potent antibacterial activity compared to standard drugs [31]. Another study by Demchenko et al. exhibited the antibacterial properties of 3-biphenyl-3H-imidazo[1,2-a]azepin-1-ium bromide derivatives against *Staphylococcus aureus* and *Cryptococcus neoformans*. The results showed that the MIC ranged from 4 to 8 μg/mL [32].

Some imidazole derivatives are currently marketed and are widely used clinically. Metronidazole is a commonly used antibiotic to treat infections caused by anaerobic bacteria, such as those found in the gut or genital tract [33]. Another example is clotrimazole, an antifungal that is used to treat fungal infections but also has some antibacterial activity against certain strains, especially Gram-positive bacteria [34].

## 4. Conclusions

The Debus–Radziszewski reaction that was utilized in this current study was proven a successful method for synthesizing the imidazole derivatives HL1 and HL2. The biological activity evaluation of these novel imidazole derivatives demonstrated that both compounds have antimicrobial activities against Gram-positive and Gram-negative bacterial strains but in different concentrations. Interestingly, the HL2 derivative showed a very high cell viability (≤5000 μg/mL) compared to the HL1 and is recommended for any future study. Based on this observation, we acknowledge that pursuing analogues through warhead substitution could indeed open a promising new direction for exploration. Additionally, more studies are required to fully understand the mechanisms underlying the antimicrobial activity of the synthesized derivatives and to optimize their therapeutic potential for clinical use. Overall, the evaluation of the imidazole derivatives with biological activity has provided valuable insights into their potential use as antimicrobial agents in future.

## Figures and Tables

**Table 1:** The minimum inhibitory concentration (MIC) of imidazole derivatives. The results are presented as mean ± SD.

Bacterial Strains	Imidazole Derivatives		Vancomycin	Ciprofloxacin
	HL1	HL2		
	(μg/mL)	(μg/mL)	(μg/mL)	(μg/mL)
*Staphylococcus aureus* (ATCC 29213)	625 ± 0.04	625 ± 0.02	0.63 ± 0.00	0.16 ± 0.00
*Staphylococcus aureus* (MRSA; ATCC 43300)	1250 ± 0.02	625 ± 0.04	0.31 ± 0.00	0.16 ± 0.04
*Escherichia coli* (ATCC 25922)	>5000	2500 ± 0.03	10 ± 0.01	0.02 ± 0.00
*Acinetobacter baumannii* (ATCC 747)	1250 ± 0.10	2500 ± 0.05	>40	0.16 ± 0.00
*Pseudomonas aeruginosa* (ATCC 1744)	5000 ± 0.04	2500 ± 0.04	>40	0.63 ± 0.10

## Data Availability

The original contributions presented in the study are included in the article; further inquiries can be directed to the corresponding authors.

## Supplementary Materials

The following supporting information can be downloaded at: https://www.mdpi.com/article/10.3390/biom14091198/s1, Figure S1: The IR spectrum for HL1 shows the distinctive peaks of C–H aliphatic at 2923 cm^−1^ and 2853 cm^−1^, C=N at 1602 cm^−1^, C=C stretch at 1513 cm^−1^ and C–O at 1283 cm^−1^; Figure S2: The ^1^HNMR spectrum for HL1 showing the ^1^HNMR, δ in ppm: (600 MHz, DMSO) δ 2.02 (3H, s, CH3), 6.81 (1H, td, *J* = 8.4 Hz, CH), 6.92 (1H, d, *J* = 8.4 Hz, CH), 7.08 (1H, d, Ar-H), 7.15 (1H, t, Ar-H), 7.2–7.37 (8H, m Hz, Ar-H ), 7.49 (2H, *J* = 8.4 Hz, Ar-H),7.52(2H, d, *J* = 8.4 Hz, Ar-H), 7.73–7.77 (2H, m, Ar-H), 7.84–7.89 (2H, m, Ar-H), 13.59; Figure S3: The ^13^CNMR spectrum for HL1 shows the ^13^CNMR, δ in ppm (600 MHz, DMSO): 109.67, 118.82, 123.55, 124.85, 126.75, 127.45, 127.65, 128.15, 128.98, 130.23, 131.14, 131.35, 132.80, 133.35, 134.06, 135.06, 135.21, 137.21, 137.76, 143.42, 154.95; Figure S4: The IR spectrum for HL2 shows the C–H aliphatic at 2923 cm^−1^ and 2853 cm^−1^, C=N at 1626 cm^−1^, C=C stretch at 1514 cm^−1^ and C–O at 1248 cm^−1^; Figure S5: The ^1^HNMR spectrum for HL2 shows the ^1^HNMR, δ in ppm (600 MHz, DMSO) δ 3.51 (3H, s, O-CH3), 6.56 (1H, td, *J* = 0.85, CH), 6.69 (2H, s, CH), 7.09.86 (1H, d, *J* = 8.5, Ar-H), 7.17 (1H, m Hz, Ar-H), 7.21–7.31 (6H, m, Ar-H), 7.46 (2H, d, Ar-H), 7.74–7.78 (2H, m, Ar-H); Figure S6: The ^13^CNMR spectrum for HL2 shows the ^13^CNMR, δ in ppm (500 MHz, DMSO): 111.18 113.76 118.32 123.34 124.34 126.72 127.35 127.73 128.28 128.62 128.91 128.98 129.04 129.31 130.42 131.34 135.05 135.25 137.10 143.87 154.94 158.77; Figure S7: The XRD spectra for HL1 and HL2 showing the distinctive peaks at 8.20°, 10.69°, 14.33°, 18.30°, 20.51°, 22.02°, 23.04° and 25.21° for HL1 and 6.28°, 7.11°, 7.90°, 11.36°, 15.96°, 20.50°, 20.70°, 21.59° and 32.33° for HL2, which indicates for their crystalline nature; Figure S8: The graphs represent the minimum inhibition concentration (MIC) assay of Imidazole derivatives (HL1 and HL2) against (A) *Staphylococcus aureus* (ATCC 29213), (B) *methicillin-resistant Staphylococcus aureus* (*MRSA*; ATCC 43300), (C) *Escherichia coli* (ATCC 25922), (D) *Acinetobacter baumannii*—(ATCC 747), and (E) *Pseudomonas aeruginosa* (ATCC 1744). All strains were treated at concentrations (5000–2.44 μg/mL). The MIC was measured at a UV absorbance of 600 nm. Negative control (only media wells) and positive control (only bacteria wells). Data shown for treated groups are the background-subtracted effect; the background is the signal of the uninoculated wells. The results are presented as mean ± SD; *n* = 3; Figure S9: The graphs represent the minimum inhibition concentration (MIC) assay of vancomycin (VAN) and ciprofloxacin (CIP) at concentrations (40–0.02 μg/mL) against (**1**) *Staphylococcus aureus* (ATCC 29213), (**2**) *methicillin-resistant Staphylococcus aureus* (*MRSA*; ATCC 43300), (**3**) *Escherichia coli* (ATCC 25922), (**4**) *Acinetobacter baumannii*—(ATCC 747), and (**5**) *Pseudomonas aeruginosa* (ATCC 1744). The MIC was measured at a UV absorbance of 600 nm. Negative control (only media wells) and positive control (only bacteria wells). The results are presented as mean ± SD; *n* = 3.

## References

[1] Kim H., Gu L., Yeo H., Choi U., Lee C.R., Yu H., Koo S. (2023). Rapid Assembly of Pyrrole-Ligated 1,3,4-Oxadiazoles and Excellent Antibacterial Activity of Iodophenol Substituents. Molecules.

[2] Tran T.N., Henary M. (2022). Synthesis and Applications of Nitrogen-Containing Heterocycles as Antiviral Agents. Molecules.

[3] Mermer A., Keles T., Sirin Y. (2021). Recent studies of nitrogen containing heterocyclic compounds as novel antiviral agents: A review. Bioorganic Chem..

[4] Kerru N., Gummidi L., Maddila S., Gangu K.K., Jonnalagadda S.B. (2020). A Review on Recent Advances in Nitrogen-Containing Molecules and Their Biological Applications. Molecules.

[5] Alghamdi S.S., Suliman R.S., Almutairi K., Kahtani K., Aljatli D. (2021). Imidazole as a Promising Medicinal Scaffold: Current Status and Future Direction. Drug Des. Devel Ther..

[6] Zhang L., Peng X.M., Damu G.L., Geng R.X., Zhou C.H. (2014). Comprehensive review in current developments of imidazole-based medicinal chemistry. Med. Res. Rev..

[7] Anderson E.B., Long T.E. (2010). Imidazole- and imidazolium-containing polymers for biology and material science applications. Polymer.

[8] Jun J., Yang S., Lee J., Moon H., Kim J., Jung H., Im D., Oh Y., Jang M., Cho H. (2023). Discovery of novel imidazole chemotypes as isoform-selective JNK3 inhibitors for the treatment of Alzheimer’s disease. Eur. J. Med. Chem..

[9] Huang W., Lu Y., Yao N., Zhang X., Wang N., Liu Y. (2024). A novel collapse strategy of zeolitic imidazole frameworks shell triggered by p-benzoquinone for the fluorescence monitoring α-glucosidase activity and screening natural anti-diabetes drug. Sens. Actuators B Chem..

[10] Solo P., Arockia Doss M., Prasanna D. (2022). Designing and docking studies of imidazole-based drugs as potential inhibitors of myeloperoxidase (MPO) mediated inflammation and oxidative stress. Biocatal. Agric. Biotechnol..

[11] Gurav S.S., Jadhav S.R., Mali S.N., Lotlikar O.A., Waghmode K.T. (2023). An efficient one-pot multicomponent, Amberlite IR120(H) catalyzed microwave-assisted synthesis of 1,2,4,5-tetrasubstituted-1H-imidazoles: Plausible mechanism and antibacterial evaluation. Synth. Commun..

[12] Raghu M.S., Pradeep Kumar C.B., Yogesh Kumar K., Prashanth M.K., Alshahrani M.Y., Ahmad I., Jain R. (2022). Design, synthesis and molecular docking studies of imidazole and benzimidazole linked ethionamide derivatives as inhibitors of InhA and antituberculosis agents. Bioorganic Med. Chem. Lett..

[13] Ali I., Lone M.N., Aboul-Enein H.Y. (2017). Imidazoles as potential anticancer agents. MedChemComm.

[14] Sharma P., LaRosa C., Antwi J., Govindarajan R., Werbovetz K.A. (2021). Imidazoles as Potential Anticancer Agents: An Update on Recent Studies. Molecules.

[15] Lee Y.T., Tan Y.J., Oon C.E. (2023). Benzimidazole and its derivatives as cancer therapeutics: The potential role from traditional to precision medicine. Acta Pharm. Sin. B.

[16] Aliwaini S., Awadallah A.M., Morjan R.Y., Ghunaim M., Alqaddi H., Abuhamad A.Y., Awadallah E.A., Abughefra Y.M. (2019). Novel imidazo[1,2-a]pyridine inhibits AKT/mTOR pathway and induces cell cycle arrest and apoptosis in melanoma and cervical cancer cells. Oncol. Lett..

[17] Wang Z., Zhang Y., Pinkas D.M., Fox A.E., Luo J., Huang H., Cui S., Xiang Q., Xu T., Xun Q. (2018). Design, Synthesis, and Biological Evaluation of 3-(Imidazo[1,2-a]pyrazin-3-ylethynyl)-4-isopropyl-N-(3-((4-methylpiperazin-1-yl)methyl)-5-(trifluoromethyl)phenyl)benzamide as a Dual Inhibitor of Discoidin Domain Receptors 1 and 2. J. Med. Chem..

[18] Yang X., Song X., Ray Banerjee S., Li Y., Byun Y., Liu G., Bhujwalla Z.M., Pomper M.G., McMahon M.T. (2016). Developing imidazoles as CEST MRI pH sensors. Contrast Media Mol. Imaging.

[19] Ghannoum M.A., Rice L.B. (1999). Antifungal Agents: Mode of Action, Mechanisms of Resistance, and Correlation of These Mechanisms with Bacterial Resistance. Clin. Microbiol. Rev..

[20] Zhou J., Cai Y., Liu Y., An H., Deng K., Ashraf M.A., Zou L., Wang J. (2022). Breaking down the cell wall: Still an attractive antibacterial strategy. Front. Microbiol..

[21] Gujjarappa R., Kabi A.K., Sravani S., Garg A., Vodnala N., Tyagi U., Kaldhi D., Velayutham R., Singh V., Gupta S., Swain B.P. (2022). Overview on Biological Activities of Imidazole Derivatives. Nanostructured Biomaterials: Basic Structures and Applications.

[22] Humphries R.M., Ambler J., Mitchell S.L., Castanheira M., Dingle T., Hindler J.A., Koeth L., Sei K. (2018). CLSI Methods Development and Standardization Working Group Best Practices for Evaluation of Antimicrobial Susceptibility Tests. J. Clin. Microbiol..

[23] Irgashev R.A., Kazin N.A., Makarova N.I., Dorogan I.V., Malov V.V., Tameev A.R., Rusinov G.L., Metelitsa A.V., Minkin V.I., Charushin V.N. (2017). Synthesis and properties of new π-conjugated imidazole/carbazole structures. Dye. Pigment..

[24] Bathula C., Ravindra M.K., Kumar A., Yadav H., Ramesh S., Shinde S., Shrestha N.K., Km M., Reddy V., Mohammed A. (2020). Microwave assisted synthesis of imidazolyl fluorescent dyes as antimicrobial agents. J. Mater. Res. Technol..

[25] Volkov M.A., Novikov A.P., Grigoriev M.S., Fedoseev A.M., German K.E. (2022). Novel Synthesis Methods of New Imidazole-Containing Coordination Compounds Tc(IV, V, VII)—Reaction Mechanism, Xrd and Hirshfeld Surface Analysis. Int. J. Mol. Sci..

[26] Kowalkowska D., Dołęga A., Nedelko N., Hnatejko Z., Ponikiewski Ł., Matracka A., Ślawska-Waniewska A., Strągowska A., Słowy K., Gazda M. (2017). Structural, spectral and magnetic properties of Ni(ii), Co(ii) and Cd(ii) compounds with imidazole derivatives and silanethiolate ligands. CrystEngComm.

[27] Uba B. (2023). Antibacterial and antifungal activities of Schiff base and its metal (Ii) complexes of Fe (Ii), Ni (Ii) and Co (Ii) derived from 2-hydroxy-1-naphthaldehyde and 2-amino-3-methylpyridine. Microbes Infect. Dis..

[28] Gratal P., Arias-Pérez M.-S., Gude L. (2022). 1H-imidazo[4,5-f][1,10]phenanthroline carbohydrate conjugates: Synthesis, DNA interactions and cytotoxic activity. Bioorganic Chem..

[29] Balandis B., Mickevičius V., Petrikaitė V. (2021). Exploration of Benzenesulfonamide-Bearing Imidazole Derivatives Activity in Triple-Negative Breast Cancer and Melanoma 2D and 3D Cell Cultures. Pharmaceuticals.

[30] Al-Soud Y.A., Al-Ahmad A.a.H., Abu-Qatouseh L., Shtaiwi A., Alhelal K.A.S., Al-Suod H.H., Alsawakhneh S.O., Al-Qawasmeh R.A. (2021). Nitroimidazoles Part 9. Synthesis, molecular docking, and anticancer evaluations of piperazine-tagged imidazole derivatives. Z. Naturforschung B.

[31] Brahmbhatt H., Molnar M., Pavić V. (2018). Pyrazole nucleus fused tri-substituted imidazole derivatives as antioxidant and antibacterial agents. Karbala Int. J. Mod. Sci..

[32] Demchenko S., Lesyk R., Zuegg J., Elliott A.G., Fedchenkova Y., Suvorova Z., Demchenko A. (2020). Synthesis, antibacterial and antifungal activity of new 3-biphenyl-3H-Imidazo[1,2-a]azepin-1-ium bromides. Eur. J. Med. Chem..

[33] Atia A.J.K. (2009). Synthesis and Antibacterial Activities of New Metronidazole and Imidazole Derivatives. Molecules.

[34] Sawyer P.R., Brogden R.N., Pinder K.M., Speight T.M., Avery G.S. (1975). Clotrimazole: A Review of its Antifungal Activity and Therapeutic Efficacy. Drugs.

