# Supplementary material for: Synthesis and Biological Evaluation of Novel Imidazole Derivatives as Antimicrobial Agents
Source: Biomolecules. 2024 Sep 23;14(9):1198. doi: 10.3390/biom14091198 (PMC11429776; doi:10.3390/biom14091198)
Supplement: Supplementary file 1 [file biomolecules-14-01198-s001.zip › biomolecules-3145122-supplementary.pdf]

Supplementary data

Synthesis and Biological Evaluation of Novel Imidazole Derivatives as Antimicrobial Agents

Huda A. Al-Ghamdi [1], Fahad A. Almughem [2], Manal A. Alshabibi [2], Abrar A. Bakr [2], Abdullah A. Alshehri [2], Alhassan H. Aodah [2], Nourah A. Al Zahrani [1], Essam A. Tawfik [2,*] and Laila A. Damiati [3,*]

[1] Department of Chemistry, College of Science, University of Jeddah, Jeddah 23218, Saudi Arabia; halgamdi4@uj.edu.sa (H.A.A.-G.); nalzahrani2@uj.edu.sa (N.A.A.Z.)
[2] Advanced Diagnostics and Therapeutics Institute, Health Sector, King Abdulaziz City for Science and Technology (KACST), Riyadh 11451, Saudi Arabia; falmughem@kacst.gov.sa (F.A.A.); malshabibi@kacst.gov.sa (M.A.A.); aabakr@kacst.gov.sa (A.A.B.); abdualshehri@kacst.gov.sa (A.A.A.); aaodah@kacst.gov.sa (A.H.A.)
[3] Department of Biological Science, College of Science, University of Jeddah, Jeddah 23218, Saudi Arabia
* Correspondence: etawfik@kacst.gov.sa (E.A.T.); ladamiati@uj.edu.sa (L.A.D.)

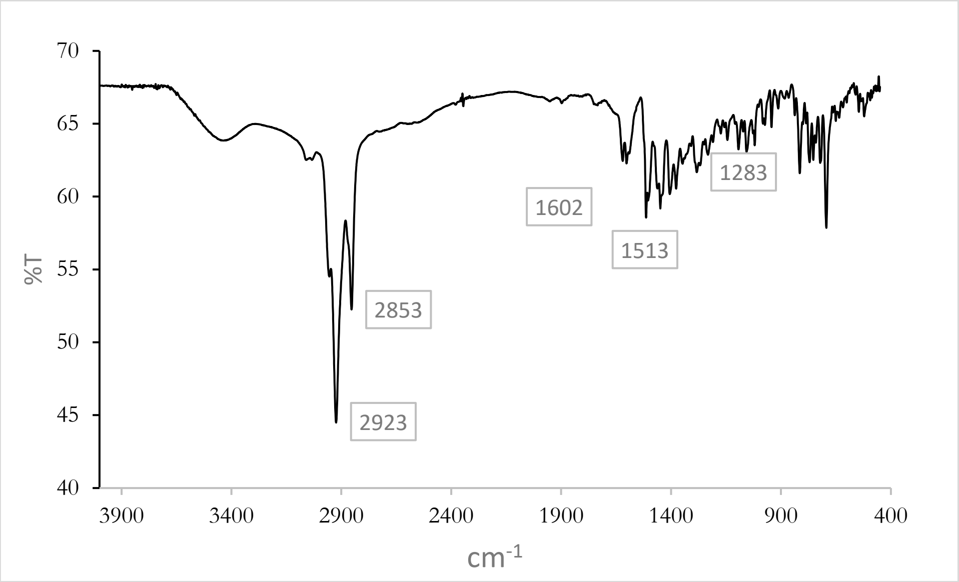

Figure S1. The IR spectrum for HL1 shows the distinctive peaks of C–H aliphatic at 2923 cm^{-1} and 2853 cm^{-1}, C=N at 1602 cm^{-1}, C=C stretch at 1513 cm^{-1} and C–O at 1283 cm^{-1}.

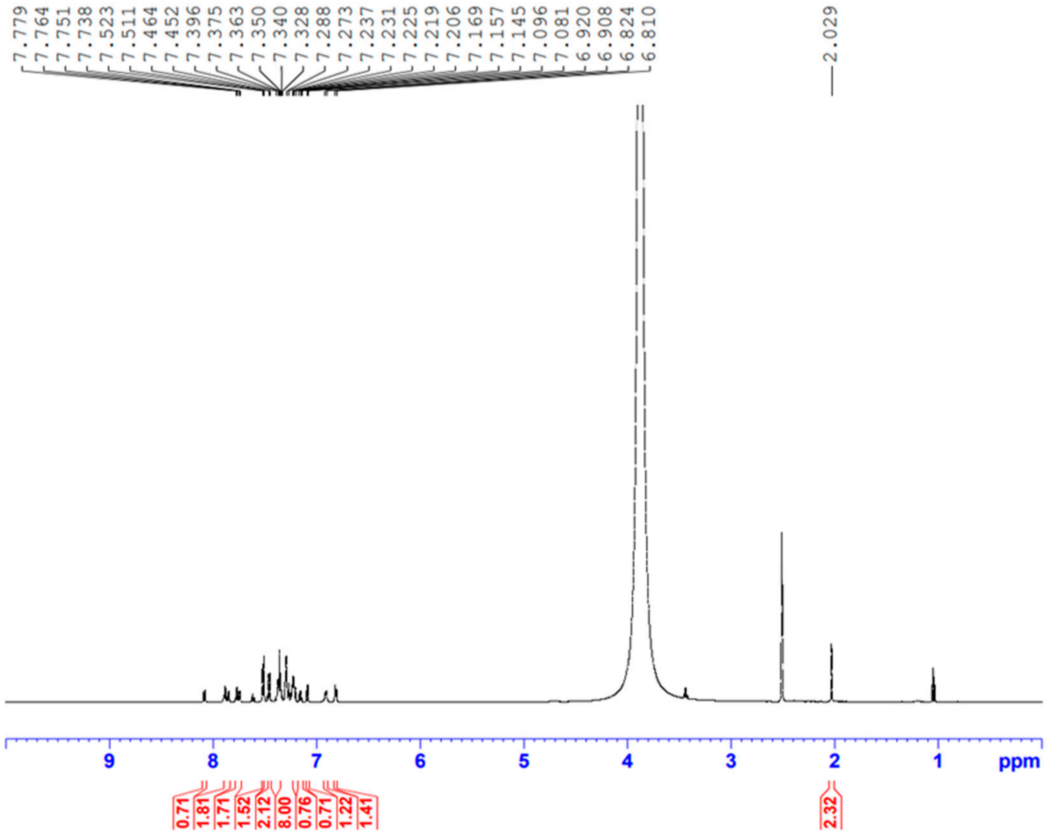

Figure S2. The ^{1}HNMR spectrum for HL1 showing the ^{1}HNMR, δ in ppm :(600 MHz, DMSO) δ 2.02 (3H, s, CH3), 6.81 (1H, td, J= 8.4 Hz, CH), 6.92 (1H, d, J=8.4 Hz, CH), 7.08 (1H, d, Ar-H), 7.15 (1H, t, Ar-H), 7.2-7.37 (8H, m Hz, Ar-H), 7.49 (2H, J= 8.4 Hz, Ar-H),7.52(2H, d, J= 8.4 Hz, Ar-H), 7.73-7.77 (2H, m, Ar-H), 7.84-7.89 (2H, m, Ar-H), 13.59.

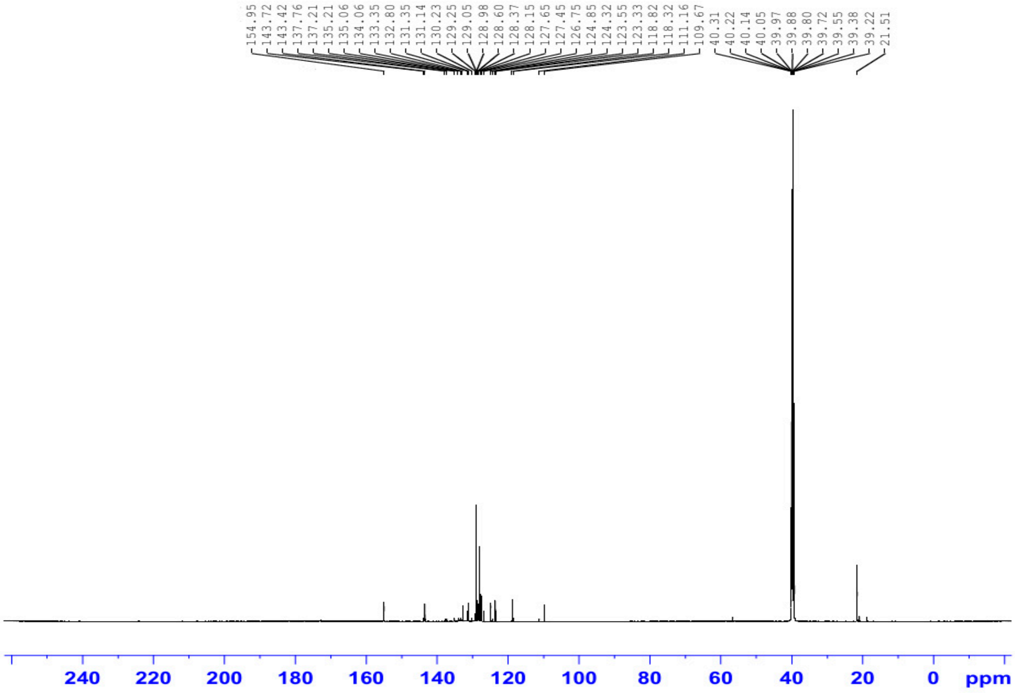

Figure S3. The ^{13}CNMR spectrum for HL1 shows the ^{13}CNMR, δ in ppm (600 MHz, DMSO): 109.67, 118.82, 123.55, 124.85, 126.75, 127.45, 127.65, 128.15, 128.98, 130.23, 131.14, 131.35, 132.80, 133.35, 134.06, 135.06, 135.21, 137.21, 137.76, 143.42, 154.95.

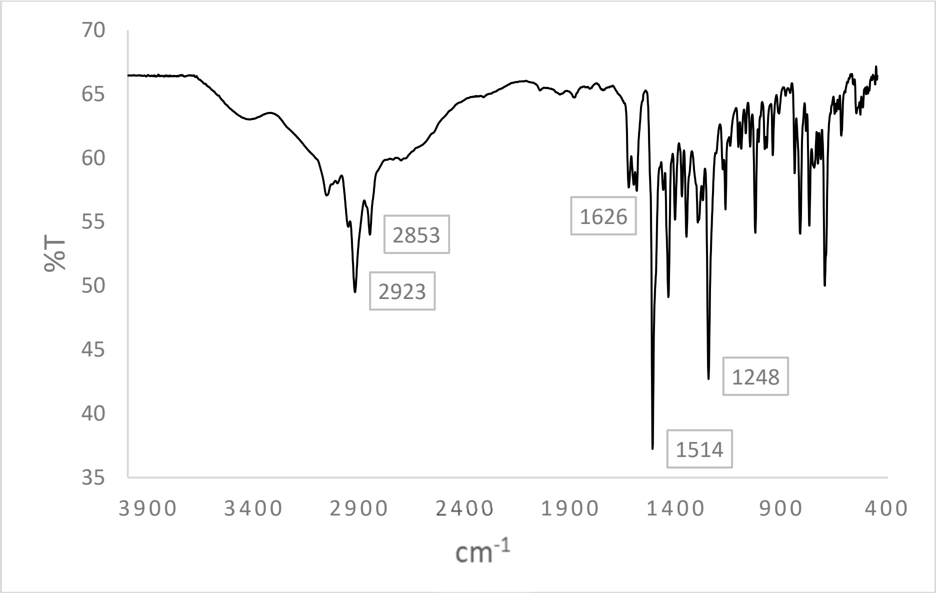

Figure S4. The IR spectrum for HL2 shows the C–H aliphatic at 2923 cm^{-1} and 2853 cm^{-1}, C=N at 1626 cm^{-1}, C=C stretch at 1514 cm^{-1} and C–O at 1248 cm^{-1}.

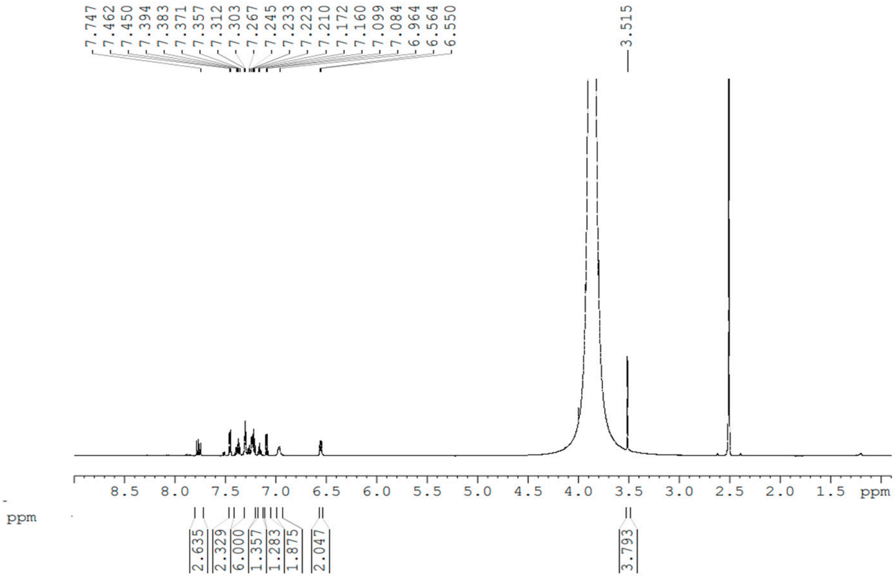

Figure S5. The ^{1}HNMR spectrum for HL2 shows the ^{1}HNMR, δ in ppm (600 MHz, DMSO) δ 3.51 (3H, s, O-CH3), 6.56 (1H, td, J=0.85, CH), 6.69 (2H,s, CH), 7.09.86 (1H, d, , J=8.5, Ar-H), 7.17 (1H, m Hz, Ar-H), 7.21-7.31(6H,m, Ar-H), 7.46 (2H, d, Ar-H), 7.74-7.78 (2H, m, Ar-H).

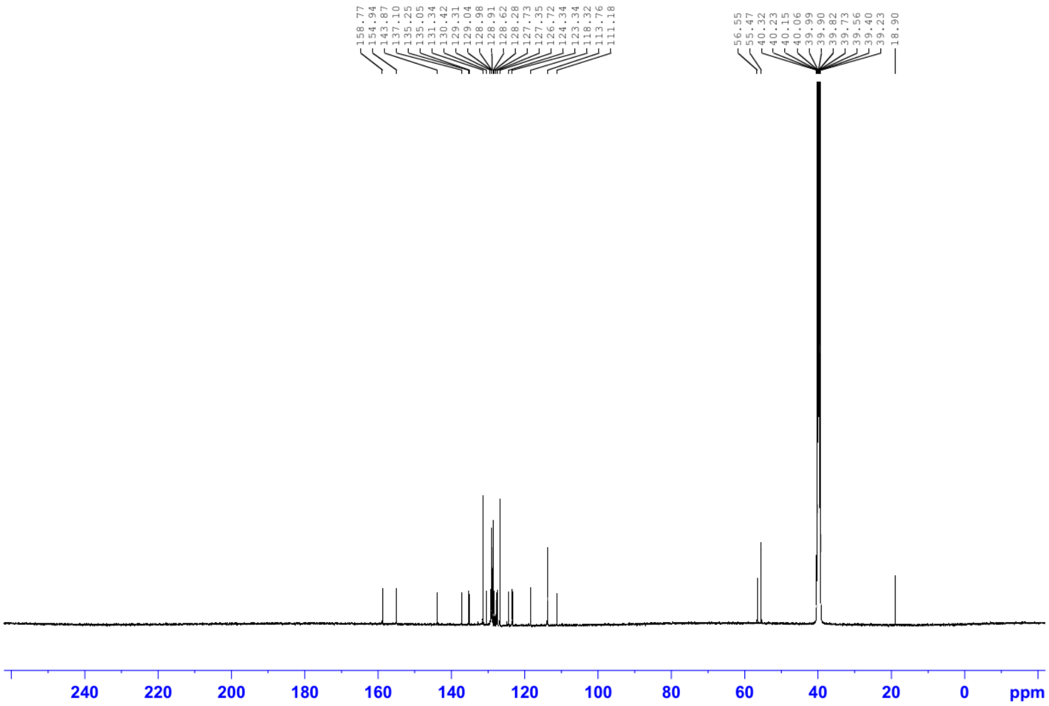

Figure S6. The ^{13}CNMR spectrum for HL2 shows the ^{13}CNMR, δ in ppm (500 MHz, DMSO): 111.18 113.76 118.32 123.34 124.34 126.72 127.35 127.73 128.28 128.62 128.91 128.98 129.04 129.31 130.42 131.34 135.05 135.25 137.10 143.87 154.94 158.77.

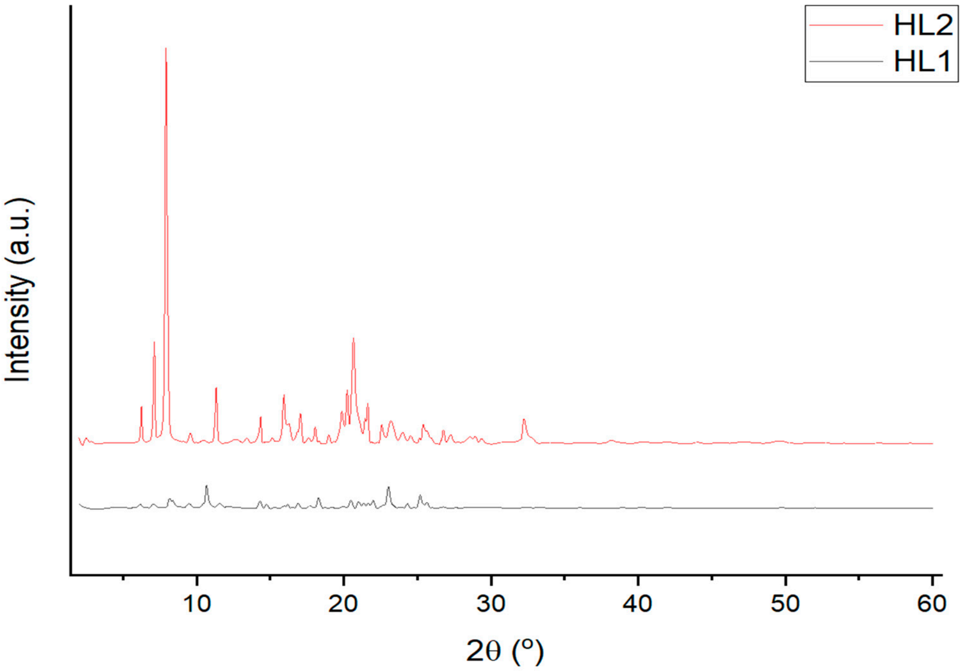

Figure S7. The XRD spectra for HL1 and HL2 showing the distinctive peaks at 8.20°, 10.69°, 14.33°, 18.30°, 20.51°, 22.02°, 23.04° and 25.21° for HL1 and 6.28°, 7.11°, 7.90°, 11.36°, 15.96°, 20.50°, 20.70°, 21.59° and 32.33° for HL2, which indicates for their crystalline nature.

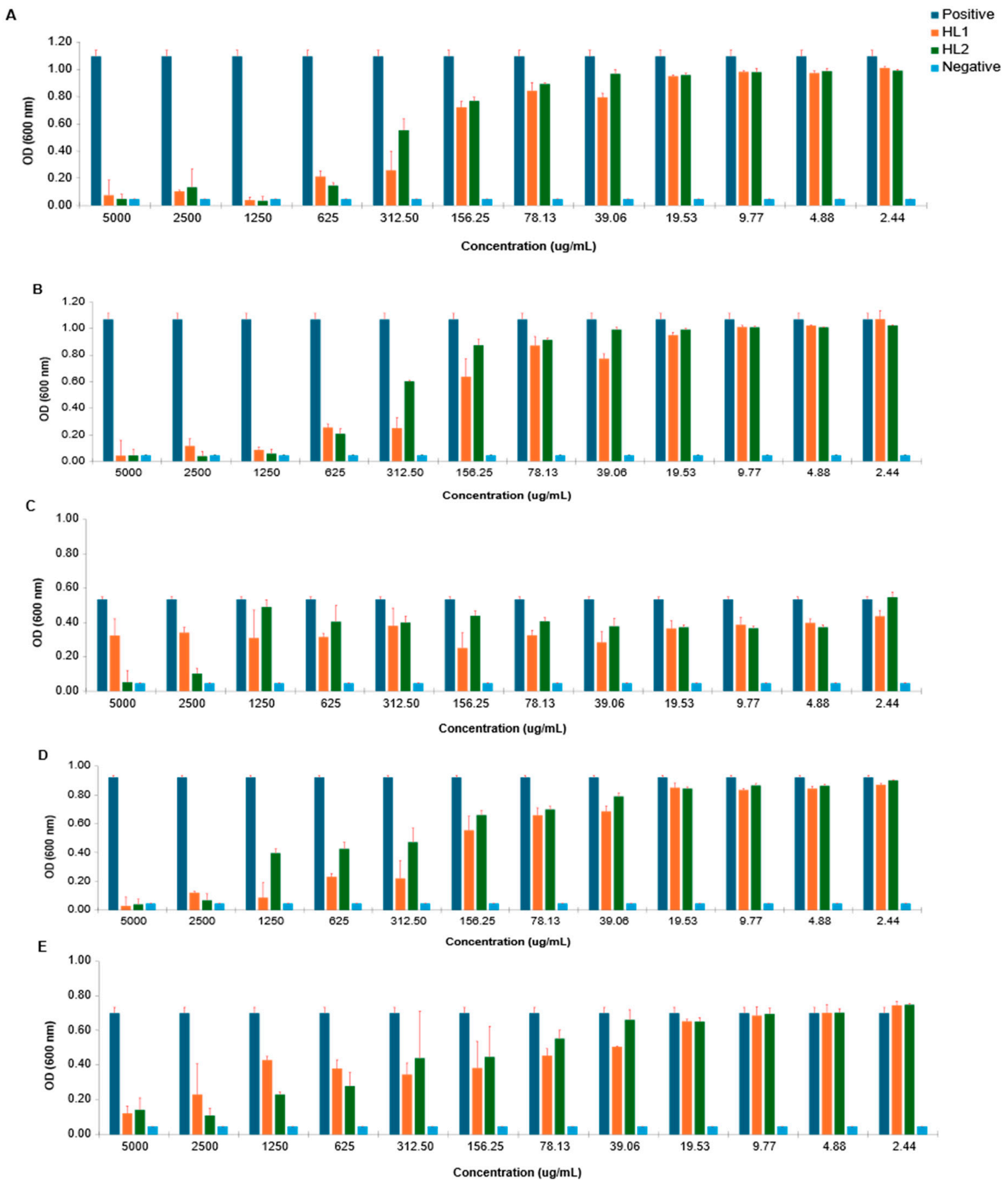

Figure S8. The graphs represent the minimum inhibition concentration (MIC) assay of Imidazole derivatives (HL1 and HL2) against (**A**) *Staphylococcus aureus* (ATCC 29213), (**B**) *methicillin-resistant Staphylococcus aureus (MRSA;* ATCC 43300), (**C**) *Escherichia coli* (ATCC 25922), (**D**) *Acinetobacter baumannii* – (ATCC 747), and (**E**) *Pseudomonas aeruginosa* (ATCC 1744). All strains were treated at concentrations (5000 — 2.44 μg/mL). The MIC was measured at a UV absorbance of 600 nm. Negative control (only media wells) and positive control (only bacteria wells). Data shown for treated groups are the background-subtracted effect; the background is the signal of the uninoculated wells. The results are presented as mean ± SD; n=3.

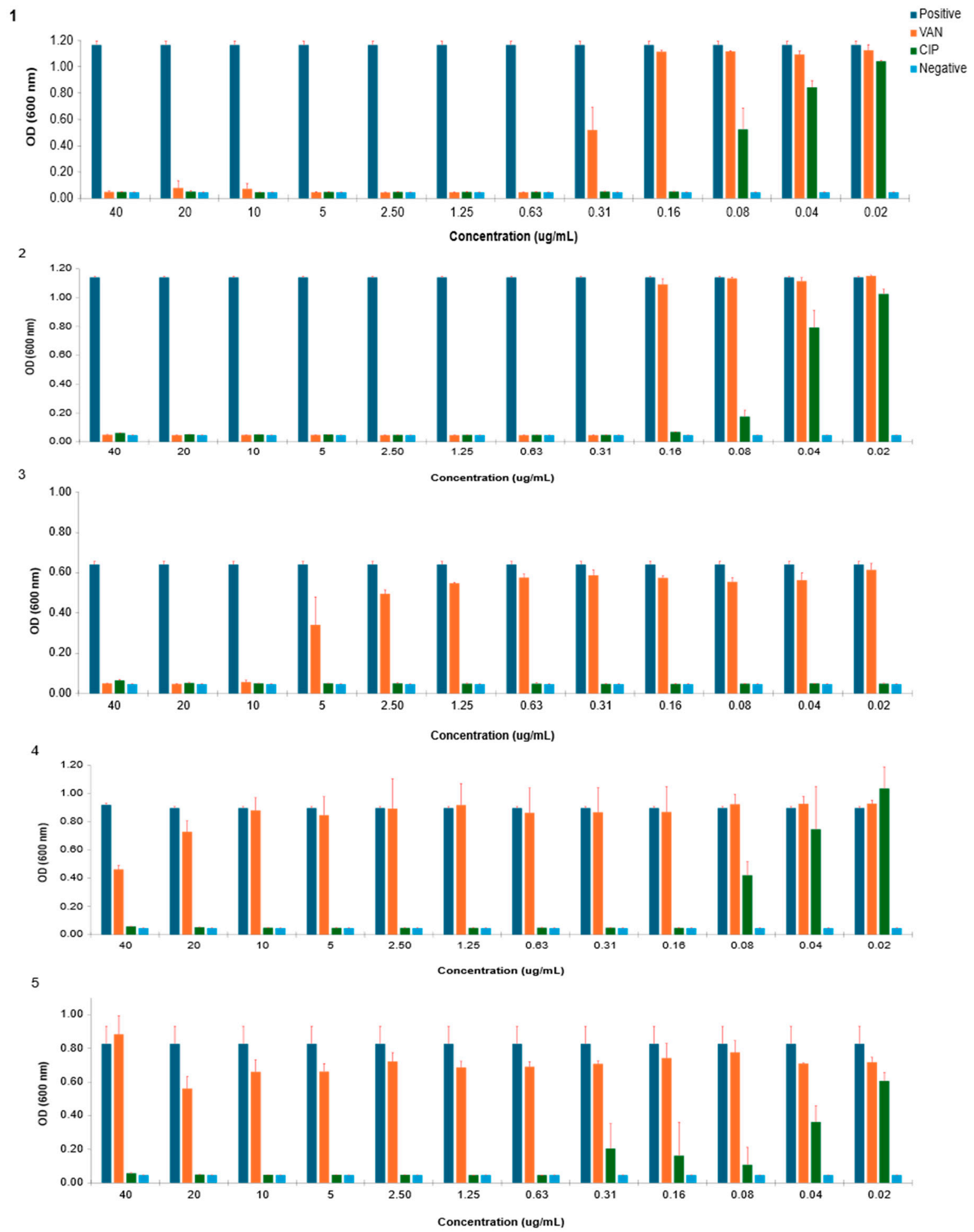

Figure S9. The graphs represent the minimum inhibition concentration (MIC) assay of vancomycin (VAN) and ciprofloxacin (CIP) at concentrations (40 – 0.02 µg/mL) against (**1**) *Staphylococcus aureus* (ATCC 29213), (**2**) *methicillin-resistant Staphylococcus aureus (MRSA;* ATCC 43300*)*, (**3**) *Escherichia coli* (ATCC 25922), (**4**) *Acinetobacter baumannii* – (ATCC 747), and (**5**) *Pseudomonas aeruginosa* (ATCC 1744). The MIC was measured at a UV absorbance of 600 nm. Negative control (only media wells) and positive control (only bacteria wells). The results are presented as mean ± SD; n=3.
